# Towards the development of a quality youth sport experience measure: Understanding participant and stakeholder perspectives

**DOI:** 10.1371/journal.pone.0287387

**Published:** 2023-07-07

**Authors:** Denver M. Y. Brown, John Cairney, Sina Azimi, Elizabeth Vandenborn, Mark W. Bruner, Katherine A. Tamminen, Matthew Y. W. Kwan

**Affiliations:** 1 Department of Psychology, University of Texas at San Antonio, San Antonio, Texas, United States of America; 2 Department of Family Medicine, McMaster University, Hamilton, Ontario, Canada; 3 School of Human Movement and Nutrition Sciences, University of Queensland, Saint Lucia, Queensland, Australia; 4 Faculty of Kinesiology and Physical Education, University of Toronto, Toronto, Ontario, Canada; 5 School of Physical and Health Education, Nipissing University, Nipissing, Ontario, Canada; 6 Department of Child and Youth Studies, Brock University, St Catharines, Ontario, Canada; Mugla Sitki Kocman University: Mugla Sitki Kocman Universitesi, TURKEY

## Abstract

Quality sport experiences may be a key underlying mechanism through which continued sport participation may facilitate positive youth development. However, what constitutes a quality sport experience for youth is poorly understood due to a lack of comprehensiveness among existing measures. This study aimed to identify the salient factors that constitute quality sport experience for youth by capturing athletes and stakeholder perspectives with a broader goal of developing a more robust quality sport experiences measure. A total of 53 youth athletes and stakeholders (i.e., parents, coaches, and sport administrators) completed semi-structured interviews or focus groups about what they felt were important aspects of a quality sport experience for youth. Inductive content analysis of the data identified four themes representing important indicators for a quality sport experience for youth: fun and enjoyment, opportunity for sport skill development and progress, social support and sense of belonging, and open and effective communication. These higher order themes were found among each of the groups that have important interpersonal relationships with athletes, as well as among athletes themselves. Each of these themes were also related to one another. Collectively, findings outline a framework to understand what constitutes a quality sport experience for youth. The Quality Sport Experience Framework for Youth will help in the development of a quantitative tool to assess this construct and enable researchers to examine how these experiences contribute to continued engagement in sport and positive developmental outcomes among youth sport participants.

## Introduction

A significant body of literature has highlighted the potential of sport to foster positive developmental outcomes for youth such as good health habits, critical reasoning abilities, social-emotional skills and social connectedness [1–3]. Considering sport is one of the most common extracurricular activities among youth [4], the development of programs that can capitalize on sports’ potential to strengthen youth development could significantly contribute to their psychological and social well-being [5]. However, participation in sport may also have adverse impacts on health and developmental outcomes for youth such as higher rates of delinquency, alcohol consumption and negative peer interactions, as well as reduced self-esteem and sense of competence [5–9]. One methodological limitation within this body of research has been the reliance on easily implementable yet crude measures that have typically been used to assess whether youth engage in organized sport or not [10, 11], which provide little information about the quality of their sport experiences and the processes by which the relationship between sport and healthy development evolves [12]. Concerns have been raised about the methodological limitations of how sport participation is currently measured, with researchers also expressing the resulting implications for understanding positive sport experiences and outcomes among youth [2, 5, 7, 8, 12]. To advance this line of inquiry, a more refined understanding of how sport participation impacts youth development is needed.

There is a notion that quality sport experiences may be a key underlying mechanism through which continued sport participation facilitates positive youth development [13]. However, the lack of a definition or framework that outlines what constitutes a quality sport experience for youth has limited progress towards addressing this knowledge gap. The absence of a well defined conceptualization of a quality sport experience may be one reason why an appropriate and comprehensive measure to assess this construct has yet to emerge, and as a result, it is not possible to unpack what may be going on in this so-called “black box” [12]. There are a variety of tools available to try and address this question (e.g., Youth Sport Experiences Survey–Sport, [14]; Motivational Climate Scale for Youth Sports, [15]; Coach Athlete Relationship Scale, [16], but none are comprehensive enough to fully capture multiple facets of youth’s experience in sport [13]). These limitations are the impetus for the present study, which will contribute to the development of the *Sport Experiences Measure for Children and Youth* (SEM:CY; [13]). However, to capture prominent experiences in sport that better predict positive and negative developmental outcomes among youth using the SEM:CY, it is important to first determine what constitutes a quality sport experience. Answering this question stands to help develop a framework from which the development of the SEM:CY can be guided.

A considerable body of literature has suggested that the experiences youth have while engaging in sport may be one of the primary reasons for continued participation [17–21]. For example, Fraser-Thomas et al.’s [1] applied sport-programming model of positive youth development suggests that appropriate design and structure in youth sport may foster positive experiences for youth athletes, leading to developmental benefits and sustained participation in sport. Conversely, when sport is inappropriately structured, it may result in negative experiences for youth athletes and can harm athletes’ developmental outcomes and minimize their desire to participate in sport. From this perspective, the quality of the sport experience is central to both the continuation of sport participation and its positive (or negative) impact on developmental outcomes.

With absence of a consensus framework to define a quality sport experience among youth athletes, Cairney et al. [13] proposed an initial evidence-informed conceptual model to serve as a starting point for developing the SEM:CY. This proposed model focuses primarily on two overarching domains–relationships and motivational climate–with subdomains for social agents such as coaches, parents, teammates, and others. Recent reviews of attrition from sport indicate that many of the same factors that are associated with drop out overlap with those identified as aspects of the quality of youths’ experience in sport [22–24]. Those that have been recognized most consistently include the motivational and social climates, which were both identified in Cairney et al.’s conceptual model, in addition to fun and enjoyment of sport.

Motivational climate refers to the degree that behaviours related to skill development (task/mastery climates) and/or winning are emphasized (ego/performance climates) [15]. The evidence suggests that when sport is structured in a manner that emphasizes skill development and mastery, experiences tend to be viewed as more meaningful [25, 26]. On the other hand, sport settings can often emphasize competition or comparisons with others (as opposed to personal development), and have been found to be strong predictor of negative sport experiences [15, 26–29].

Social climate refers to the interpersonal relationships amongst the team, which, given their roles in youth sport, also extends to coaches and parents in this context. Previous research suggests the caring and supportive behaviours displayed by peers, coaches, and parents are a significant contributor to youth sport experiences [30–35]. Athletes who build a positive rapport with their peers and coaches generally report more positive experiences [35–37]; whereas, negative interpersonal dynamics detract from the experience. Finally, fun and enjoyment has been identified as important aspects of a quality sport experience, particularly during childhood [21]. Thus it is no surprise that many sport organizations will market enjoyment in their mission statement, and that an overall lack of enjoyment can explain the precipitous decline in sport participation during adolescence [38, 39].

The existing research provides a foundation for helping to define a quality youth sport experience; however, to capture the essence of a quality sport experience using a single comprehensive and parsimonious measure, more empirical investigation is needed to better understand the core domains and how they relate to each other within a broader construct. Qualitative research methodologies represent one approach that can produce rich data to reveal the complexity of what constitutes a quality sport experience for youth. Through taking an interpretive approach, qualitative research can ultimately generate a comprehensive description and interpretation of phenomena through the voices of the participants and the reflexivity of the research [40]. Conducting rigorous qualitative inquiry is necessary to develop an integrative understanding of the factors that define a quality sport experience; and in turn, will help to inform the development of the SEM:CY.

Additionally, further clarification is required to understand the key factors that help to facilitate a quality sport experience for youth athletes. Cairney et al.’s [13] conceptual model of sport experiences for children and youth acknowledges the social environment as an instrumental condition for supporting quality sport experiences. This aligns with previous research showing peers/teammates, parents and coaches all play influential roles in youth’s sport experiences [2, 41–44]. Each of these stakeholders likely have their own unique views about what may facilitate (or hinder) a quality sport experience for youth and little research has investigated their points of view. Youth athletes themselves may be less cognizant of how others around may be trying to help create a quality sport experience, and it would be beneficial to also explore the understanding of what quality sport experiences means to different stakeholders for youth sport participants. Although previous work of positive youth development (e.g., applied sport-programming model) provides a clear connection between sport structure and positive experiences for youth participants, the overarching body of literature lacks an empirically informed framework from which future studies can comprehensively determine whether quality sport experiences underly the relationship between sport participation and positive youth development.

The purpose of the present study was to identify the salient factors that constitutes a quality youth sport experience with the consequent goal of developing the SEM:CY measure. This will be accomplished by capturing not only youth’s perspectives, but also with the integration of perspectives from stakeholders known to contribute to youth’s sport experiences. This aspect of the SEM:CY project represents the qualitative component of measurement development: establishing a framework to assess this construct will be instrumental for developing a more complete quality sport experience measure for youth.

## Method

### Study overview

In this study we adopted an interpretive descriptive approach [45] rooted in a constructivist paradigmatic position, which aims to understand and interpret meanings of events that are created through construction and/or reconstruction of individuals’ lived experiences [46]. Adopting a constructivist paradigm involves a relativist ontology and a subjectivist and transactional epistemology [47]. Ontologically, a constructivist approach holds the assumption that subjective realities are constructed as a result of multiple mental reconstructions that are socially and experientially based [46]. Epistemologically, the constructivist paradigm assumes that knowledge is constructed through transactions between the researcher and the participant(s) [46]. Therefore, throughout the research process, there can be no separation between the researcher and the participants, which ultimately leads to co-creation of findings between the knower and the known [48].

### Participants and recruitment

Purposeful sampling was used to recruit participants for this study [49]. Participants were recruited using a variety of methods. We recruited youth athletes between the ages of 10 to 18 years old (*M*_age_ = 13), as well as parents, coaches, and sport administrators from a variety of individual and team sports across Canada. Research staff approached coaches and administrators at multiple events (e.g., sport conferences, banquets) and provided those interested with contact information. Interested coaches and administrators were also asked to share the study information with their athletes and parents of their athletes. All interested participants contacted the research staff through email and a mutual time was set up to for either an interview or focus group to occur. A letter of information and consent form was emailed to each participant, which they were required to sign, scan, and email back to the research team prior to their interview. All participants were offered a $20 CAD gift card as remuneration for their participation. Informed written consent was obtained for youth participants as well as from parents and guardians prior to the start of each interview. The University of Toronto Research Ethics Board approved the protocol for this study. All procedures were conducted in accordance with the Declaration of Helsinki.

Our total sample (*N* = 53) consisted of 15 youth (five females and ten males), 18 parents (twelve females and six males), 20 coaches (eight females and twelve males), and 9 administrators (two females and seven males). Nine participants self-identified as occupying multiple roles (e.g., a parent who was also a coach), which accounts for the discrepancy between our subgroup total and the total sample. Youth participants were included if they had participated in a competitive or recreational organized sport (i.e., with a coach) within the past year. Similarly, parents were required to have a child (10 to 18 years old) who had participated in an organized sport within the past year. Coaches and administrators were required to have a minimum of one year of coaching/administration experience in an organized sport/organization at any level. Participants across all categories represented a wide variety of sports including figure skating, soccer, volleyball, ball hockey, karate, track and field, baseball, dragon boating, football, softball, swimming, basketball, synchronized swimming, judo, and ice hockey. To maintain anonymity, participants were assigned a number (1–53). In the results section, coaches are identified as C, parents are identified as P, youth athletes are identified as Y, and sport administrators are identified as A. If a participant self-identified with multiple groups, this was noted in the results section. For example, P/C-23, would refer to participant 23 who is a parent and a coach.

### Data collection

Parents, coaches, athletes, and administrators participated in semi-structured interviews or focus groups [50]. Interviews and focus groups were either conducted in person, remotely using cloud-based video conferencing, or over the phone. To maintain confidentiality, all interviews were audio recorded using a Sony PX370 Mono Digital Voice Recorder, regardless of it they were completed in person or remotely.

One interview guide was used for parents, coaches, and administrators, and a separate interview guide was used for youth participants. The interview guides (available upon request) were broad in nature to allow for open and unbiased discussions as to what the participants felt were important aspects of a quality sport experience [51]. Topics were discussed until participants expressed that they had adequately discussed the relevant theme before moving on to the next topic. Youth interview topics included: (a) their favorite things about playing sport, (b) key members of a youth’s sport experience (i.e., parents, coaches, teammates) and their interpretation of how they influence a youth’s experience in sport, (c) how important the youth felt winning was to parents/coaches/teammates, (d) how important they felt helping them play better was to parents/coaches/teammates, and (e) things they have learned through sport. The interview topics for parents, coaches, and administrators involved similar questions about the influence of others on a youth’s sport experience (e.g., Can you speak about how spectators, coaches, parents and/or teammates impact a youth’s experience in sport?), with additional questions about the concept of a quality sport experience including: (a) what does a quality sport experience mean to you?, (b) what do you feel are some key ingredients of a quality sport experience?, and (c) what does it look like when a youth is having a quality sport experience?

In total, 38 individual interviews and six focus groups were completed. Focus groups included youth (3 groups x 2 participants and 1 group x 3 participants), administrators (1 group x 4 participants) and parents (1 group x 2 participants). If a parent and a child from the same family were being interviewed separately, confidentiality remained so that what the youth discussed was not brought up in their parent’s interview and vice versa. Individual interviews ranged from 17 to 27 minutes (*M* = 23 minutes) for youth, 20 to 58 minutes (*M* = 39 minutes) for coaches, 20 to 63 minutes (*M* = 42 minutes) for parents, and 17 to 55 minutes (*M* = 37 minutes) for administrators. Focus group interviews ranged from 23 to 42 minutes (*M* = 31 minutes) for youth, whereas the focus group with parents was 52 minutes in duration and the focus group with administrators was 57 minutes. These de-identified data are available upon request. An institutional research ethics board approved the protocol for this study.

### Data analysis

The transcripts were analyzed using inductive content analysis, which is a systematic approach for coding, categorizing, and analyzing written, verbal, or visual communication messages [52, 53]. Following the procedures outlined by Elo and Kyngäs [52], researchers began the preparation phase by reading through participants’ data several times to become immersed in the data, and to further make sense of the participants’ accounts and their overall story. In the organization phase, researchers engaged in open coding of the data by creating headings that highlighted participants’ narratives about their experiences in youth sport. For example, ‘having fun’ was used as a heading to highlight participants’ enjoyment in youth sport. Headings were then grouped together to create sub-themes. For instance, headings such as ‘Friendship’ and ‘Socialization’ were grouped together to create a sub-theme that focused on the participants’ socialization in youth sport. The analysis process continued by grouping the sub-themes under higher order themes to provide a means for understanding and describing the phenomenon, and to generate knowledge. For example, sub-themes focusing on participants’ positive experience in youth sport were grouped together to develop the ‘Fun and Enjoyment’ theme. Themes were created based on researchers’ interpretation of the sub-themes and their ‘belonging’ to a particular group. In the abstraction phase, higher order themes were labeled using content-characteristic terms, and a general description of the research topic was formulated.

## Results

Across all interviews and focus groups, four themes were identified as representing important indicators for a quality sport experience for youth: fun and enjoyment, opportunity for sport skill development and progress, social support and sense of belonging, and open and effective communication. These higher order themes were found among each of the groups that have important interpersonal relationships with athletes, as well as among athletes themselves. Each of these themes were also related to one another. Each theme is discussed in detail below.

### Fun and enjoyment

When athletes were asked about what contributes to a quality sport experience, their responses consistently identified the hedonic factors of fun and enjoyment as most important. For example, Youth-10 said: “I like the fun of it. I just like playing.” Parents generally reiterated the views of their children in that fun is central to a quality sport experience: “I think [a quality sport experience] is one that’s easy for them to like or easy for them to enjoy” (P-16). One parent noted that the conversations occurring during the car ride home help to illustrate the importance of fun and how it contributes to continued engagement in sport:

“We know right away as parents if they’re not having a good experience because you hear about it in the car ride home and they don’t want to go back next time. When things are good, they’re excited, they have fun, they talk about it and they want to go back.”

(P-12)

Given that several parents were also coaches, it wasn’t surprising that coaches expressed a similar notion: “You want them to enjoy themselves, enjoy the game, have fun. At the very least they need to have fun because otherwise why are they coming out” (C-26). This quote also reflects coaches’ awareness surrounding why some athletes may disengage from sport as a result of a lack of fun and enjoyment. At the same time, coaches acknowledged their responsibility in creating an environment centered around fun and enjoyable:

“I think [coaches] have a huge impact–positively, negatively, every which way. I think they’re probably the biggest influencers of the experience via the environment they create–if it’s enjoyable and fun, that kind of thing.”

(P/C-53)

In another example, Coach/Administrator-51 described the coach’s role in tailoring sport programming to be an enjoyable experience that will have a positive influence on sport participation across the life course:

“Kids have to enjoy sports in order to become life-long participants, which is very important to me, and that enjoyment has to be built into the programming and the coaching.”

(C/A-51)

Collectively, these findings suggest the fun and enjoyment within their sport is an integral and central component of a quality sport experience for youth. Participants’ views were consistent in that coaches are in a unique position in which they can structure the sport programming to ensure athletes have fun and enjoyable experiences.

### Opportunities for sport skill development and progression

The next salient theme identified were the opportunities for developing sport skills and continued progression. Being given the opportunity to learn and practice new sport skills was found to be critical for sport skill development from the athlete’s perspective. For instance, when asked what some of their favourite activities were that they did in practice, Y-45 answered:

“Learning new techniques … Practicing things that you’re able to practice, learning.”

Speaking from a youth perspective, one parent mirrored the above sentiment, but also reported a need for skill development to be noticeable by athletes themselves:

“I would say if it was sort of from the athlete’s perspective–being given that opportunity to develop skills … Knowing that my time is not being wasted and that I am making progress within the sport.”

(C/P-53)

Another parent felt that seeing the tangible progress being made was a key to continued engagement for their child in the sport, believing that those youth participants who fail to see improvement are most likely to disengage:

“And I think for them it also helps them keep interested if they’re feeling like they’ve moved forward. I think if you felt stagnated, kids lose interest pretty quickly and I think that would add to it.”

(P-17)

Consistent with findings for fun and enjoyment, athletes and their parents viewed coaches as the main social agents responsible for providing athletes with opportunities to develop their sport skills:

“Well, I think the coaches are the primary teacher on the field. And for kids to develop it’s important that coaches are constantly teaching them and re-teaching skills in order for them to develop as a player”

(P-13)

Simply learning new skills, or the continued practice of acquired skills, may not be enough to create a quality sport experience for some athletes alone. The general sentiment among athletes were that they count on their coaches to provide appropriate practice contexts that will not only challenges them in skill develop, but one that will allow them to make progress towards reaching their potential: “So, my coach right now I really like because he pushes us to do better” (Y-18). The quote highlights the instrumental role of coaches in addressing factors that contribute to a quality sport experience, such as creating a mastery climate through seeing up appropriate challenges. In fact, coaches are in a unique position given that they have perhaps the greatest understanding of an athlete’s skill development, enabling them to work with and to communicate with an athlete about how their skills have progressed. C-34 acknowledged that at times, athletes may not recognize the improvements they have made, and this is where a coach can have significant impact on an athlete’s perceptions of their experience through providing positive feedback about their development:

“You need to show it to them and say look [name] this is what you were at the beginning of the year at this section of the skill, you’ve now improved your under water kicking skill by this much. And they’re going to be like wow I didn’t even notice that. And that makes them feel good.”

Most coaches acknowledged that providing feedback on progress was an important role they held, and that this was a key part of developing a quality sport experience for youth athletes.

Beyond contributing to skill development at the individual level, athletes also recognized the importance of coaches for providing opportunities for all athletes to develop their skills, thus ensuring that the team improves as a collective unit. Understandably, this theme was only described in team sports; but nevertheless, athletes felt that coaches who emphasized the importance of teamwork, particularly through teaching them to play off each other’s talents, helped facilitate success and ultimately a quality sport experience:

“Oh yeah. I think it’s important that we all develop together, and we all improve as a unit and not just like certain players. Like all going off and being an individual player when we’re all on a team; so we all have to develop together so we can like learn each other’s strengths and weaknesses so that we can play off of each other on the field.”

(Y-18)

### Social connections: Social support and sense of belonging

Positive social connections were also identified as a salient theme to represent an important dimension of a quality sport experience. Among the youth sport participants, it was clear that the peer relationships helped to foster a sense of belongingness: “I like being on a team and feeling like a part of something. And working together to accomplish a goal” (Y-18). The social connections were important for many, as some participants even indicated a switch in sport programs to seek greater social connections: “So I stopped playing basketball because I don’t know, it just wasn’t as enjoyable I guess; and then went to volleyball where I could relate more with other people” (Y-21).

Other athletes spoke about how the supportive nature of the sport environment contributes to the overall experience:

“It just feels nice because getting a goal with your teammates and they’re happy for you, it just feels really good because everyone is supporting you for that one moment and then you get to support someone else in the next.”

(Y-7)

Youth athletes themselves acknowledged that the opportunity for social connections was likely a primary reason for why their parents had signed them up for sports to begin with:

“I think the reason why parents put their kids in sports so young and try to get them involved is for that team kind of feel. Like to get that kind of extra almost family like environment and those kinds of friendships to develop.”

(Y-23)

Similarly, parents consistently identified positive social connections and support when discussing social aspects that contribute to a quality sport experience:

“But having teammates that you feel supported by is only going to help you want to come back and help you succeed in the sport as well. So you have teammates that you get along with, coming to practice is far more fun

(P-9).”

These social elements were also related to athletes’ desire to continue engaging in sport:

“At those ages, like 10, 11, 12, 13, all the way up top 18, I think your teammates can have a huge impact on you, those are the social years. And so if you have really great friends that are part of your game or part of your program or part of your team, I think that’s key for you wanting to stay in sport and continue on.”

(P-48)

The coaches’ views were consistent with those of parents. Coach/Administrator-51 highlighted the positive impact a sense of belongingness can have on sport experiences for youth:

“I think very positively. Kids want to be on teams where they feel accepted, can enjoy the company of other players on the team, can develop social relationships, can cheer together or they can cry together, can discover things together, can explore together, and can be creative together.”

Another coach elaborated on this line of thinking in that support behaviours manifest through these strong social bonds:

“I think social [connections] is a huge, huge, part of the experience of an athlete… Absolutely. I think they are integral. It’s that bonding. It’s that cohesion. It’s that, that immediate support network that you don’t have to force friends. It’s one of the social enamors of life. Positive teammates, absolutely into positive experiences… I think also connecting with their teammates is a sign of enjoyment.”

(C-15)

Coaches were again identified as the social agents responsible for creating an environment where establishing social connections was possible. Not unlike the other themes, coaches were well aware of their responsibility in creating positive social dynamics within the sport environment. One coach explained how this can be accomplished through establishing connections with their athletes: “They’re more inclined to listen to you and to care the more you show you care. You actually take an interest in their lives instead of just show up, do these drills and go home (C-26).” Coaches also expressed how the benefits of establishing connections extended beyond their relationships with athletes. The importance of a supportive environment was best showcased by C-19, who acknowledged the need to create an environment where athletes are supportive of one another in pursuit of a greater goal:

“I think having a group of teammates that as a minimum, you don’t have to get along outside of practice hours, you have to have understand how to get along and support each other during those practices, competition hours. And I think that the onus for that kind of rests on the coach.”

Together, it was widely viewed that a supportive environment in which youth participants can establish a sense of belongingness is an important contributor to quality sport experiences. Youth athletes, parents and coaches all recognized the formative role coaches play in structuring this type of environment.

### Open and effective communication

The final theme that was identified as contributing to a quality sport experience was related to open and effective communication. This theme, however, was most salient among parents of youth sport participants rather than among the athletes themselves. Many parents talked about the importance of open lines of communication between coaches and athletes, as well as between the coaches and themselves as parents. Perhaps because of their peripheral involvement, parents were often highly aware of the effect that strong communication between coaches and athletes could have on an athlete’s experience. When asked about what contributes to a quality sport experience, Parent-13 expressed that open and effective communication is particularly influential for athletes who are just beginning to learn a new sport:

“I would say positive communication. Questions are being answered. That’s probably it…. I know my daughter on the field always asks questions especially when she’s new to a sport and it is important for [the coach] to clearly answer them I guess.”

Another parent suggested that it was critical for coaches to have an open channel of communication with athletes:

“If that level of communication isn’t open between the coach and the athlete, where the athlete and the coaches are comfortable talking about it, then you’re going to lose an athlete for it.”

(P-19)

One parent identified how poor communication between coaches and athletes could negatively affect an athlete’s experience, and could ultimately lead to drop-out:

“I think a lot of times [the coaches] were speaking at a level that my kids couldn’t understand. Or the coaches had expectations of my kids that my kids were too young to question. So yeah if they got frustrated with that coaching experience, on the way home that day I would definitely learn that they don’t want to go back.”

(P-12)

Many parents also stated that they felt it was important for coaches to have open lines of communication with them too. Through declaring their expectations and goals for the upcoming season to both athletes and their parents, coaches can create an environment that fosters quality sport experiences:

“If the coach is upfront and says this year the goal is to have fun and learn new skills and everybody will be getting equal playing time. Just being able to communicate those goals and expectations I think makes for a positive experience for the players and the parents because then you know what the season is about. And then you maybe can understand why they’re doing certain things. But just knowing those expectations can make or break a season with the team.”

(P-6)

It was apparent that coaches needed to be flexible in how they communicate when providing athletes with opportunities to develop skills in a manner that suits their needs. One participant spoke about the critical role that communication had on opportunities for sport skill development and learning about their progress. Youth-3 stated:

“So the key ingredients obviously would have to be communication; and when I say communication, I mean there are different types of learning styles for these kids. Some are visual learners. You know so it’s learning to adapt to those learning skills to, to assist the player itself to understand what their expectations are.”

As with the other themes, communication itself does not independently contribute to a quality sport experience, but it is an important aspect that can have an effect on the other themes identified, and ultimately contribute to whether a child would want to continue participating in that sport or not.

## Discussion

**Fig 1:**
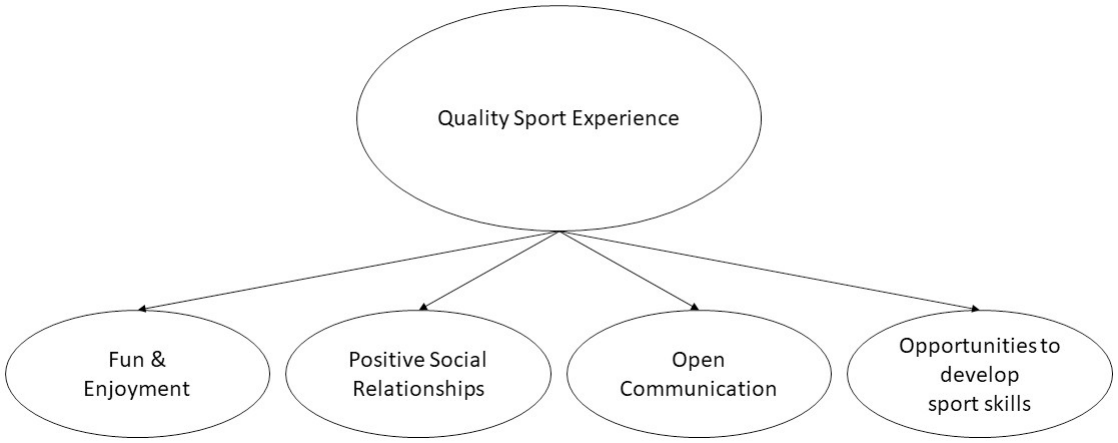
Proposed quality sport experience framework for youth.

The present study sought to uncover the salient factors that contribute to a quality sport experience for youth sport participants and represents the initial phase of the SEM:CY development [13]. Despite gaps in current understanding regarding the processes by which sport participation impacts youth development, there is a growing notion that quality sport experiences play an influential role in this relationship [13]. Together, four higher level themes were identified as important indicators for a quality sport experience in youth athletes: fun and enjoyment, opportunity for sport skill development and progress, social connections, and open and effective communication. These higher order themes were identified among each of the groups that have important interpersonal relationships with athletes, as well as among athletes themselves. Each of these themes were also related to one another. That is, the creation of a fun and enjoyable sport context was influenced by the way coaches communicated with their athletes, and how sport practices are created to help athletes acquire, develop, and progress in terms of specific sport skills. Furthermore, it was clear that sport programming should be done within the backdrop of a socially supportive environment which enables athletes to develop their sport skills while feeling a sense of belongingness. Collectively, these findings outline a novel framework that needs to be captured as quality sport experience for youth, providing a roadmap in the development of the SEM:CY (see Fig 1).

Taken together with existing evidence, results derived from our analysis of 38 interviews and 6 focus groups generally align with previous literature examining determinants of quality sport experiences among youth. Fun and enjoyment for example, were identified to be a central component of a quality sport experience, which was linked with each of the other domains we observed–a finding that aligns with Visek et al.’s [21] fun integration theory of youth sport participation. The views expressed by our participants also supported Cairney et al.’s conceptual model and previous work that has demonstrated the importance of a supportive environment in which athletes can develop meaningful relationships with not only their peers, but their coaches as well [30, 32, 35–37]. Athletes acknowledged a strong sense of belongingness contributes to their enjoyment of the sport experience, which further highlights the interdependency among the domains we identified. The importance that participants placed on having opportunities to develop their sport skills and observe progress also aligns with existing research that has investigated motivational climates. Specifically, participants did not identify winning or competition (i.e., ego/performance climates) as critical aspects of a quality sport experience, but rather, task/mastery climates were viewed favourably for facilitating meaningful experiences [25–29]. Overall, this study represents the first comprehensive investigation of salient factors impacting youth sport experiences and capturing multiple viewpoints permitted deeper insight into specific aspects of sport that need to be measured in order to determine whether quality sport experiences are an underlying mechanism by which sport may contribute to positive youth development.

Although not an original aim of the study, it became readily apparent throughout the interviews that coaches were positioned as being the primary social agent for structuring the sport environment to promote quality experiences. This was acknowledged by the athletes themselves as well as their parents who consistently recognized their responsibility in this regard. Previous research demonstrates that a fun and enjoyable environment is a key motivator of continued sport participation for youth [54, 55]. The interdependence between fun and enjoyment with other domains was evident in interviews with coaches. For example, they highlighted the significance for enjoyment of sport programming tailored to an athlete’s skills (i.e., promoting progress through incremental challenges). Moreover, several coaches expressed how social elements such as poor communication and opportunities to be around friends were related to athlete’s enjoyment of sport and ultimately, whether they had a desire to continue engaging in sport. Being with friends is an important social motivator for youth sport participation [56], whereas emerging evidence from studies examining conversations between parents and their children on the way home from sporting events has shown how poor communication with coaches can drive kids away [57, 58]. Collectively, these findings fit within the personal relationships and appropriate settings gears of Côté et al.’s [59] personal asset framework of sport participation, which are proposed to interact over time to influence sport participation and positive youth development.

Findings from the present study address a critical knowledge gap in the literature regarding what constitutes a quality sport experience, presenting a novel framework for operationalizing quality sport experiences for youth. Our proposed quality sport experience framework for youth shares considerable overlap with the conceptual model proposed by Cairney et al. [13] in that social interactions and opportunities for developing sport skills are considered key domains that contribute to a quality sport experience, although this current framework builds upon their model by presenting the importance of two additional domains related to open communication and fun and enjoyment. Evidently, multiple facets need to be considered when developing the SEM:CY to provide a comprehensive representation of this construct. Recent work in parasport also lends some important insight. Working with multiple stakeholders from the parasport community, Evans et al. [60] developed the Quality Parasport Participation Framework. At its core is quality experience, which was defined as “a feeling state involving satisfaction and enjoyment, based on an athlete’s appraisal of whether parasport experiences satisfy one or more of their own sport values and needs” [60]. Six interrelated elements were identified as key determinants of a quality experience: belongingness, autonomy, challenge, mastery, engagement, and meaning. This framework also identified the importance of the social and physical environment for fostering conditions that support quality sport experiences. Some elements, such as the need for positive relationships and developmentally appropriate challenges that enable individuals to master skills, were apparent within both frameworks. However, disparities appear to exist across these populations, which is an important finding because it showcases that population-specific frameworks are needed and a one-size-fits all approach to understanding what constitutes a quality sport experience is not warranted. In sum, collecting new data from multiple perspectives enabled us to refine the conceptual framework put forward by Cairney et al. and the broader themes that were identified will help ensure the SEM:CY captures the many facets that contribute to quality sport experiences for youth.

Although the present study addressed key gaps in our current knowledge, it is not without limitations. First, our sample of youth only consisted of individuals currently engaged in sport, thus our findings fail to reflect the experiences that may have driven youth out of sport. Youth who are participating in sport may have different sport experience perspectives than youth who may have discontinued participation. Potential differences in sport experiences based on whether youth are still participating in sport or not is an especially important consideration during adolescence when substantial attrition from sport occurs [39]. A second limitation is related to generalizability, as the current sample included primarily younger youth athletes that were involved in recreational pursuits, which may help to explain why potential themes related to competition and winning were not readily identified as important aspects of a quality sport experience. Finally, although our aim was to capture multiple viewpoints to understand what constitutes a quality sport experience for youth, which is a strength of this study, several participants held multiple roles (e.g., parent and coach), making it difficult to tease apart these perspectives. The dualistic nature of stakeholder roles may be especially challenging in youth sport research given that the infrastructure of youth sport often relies on volunteer efforts (although this differs across countries), which is most likely to come from parents of participants. Despite attempting to gain more in-depth perspectives from individuals serving different roles within sport organizations (i.e., coach vs. administrator), most of what was shared appeared to be in line with coaches’ perspectives (e.g., on the ground during sport practices or competitions), rather than more logistical or organizational considerations at the administrative level (i.e., economics, accessibility to sport venues, opportunities or travel for competitions, etc.).

Overall, findings from this qualitative investigation suggest that youth sport participants and important stakeholders viewed fun and enjoyment, opportunities for sport skill development and progression, social opportunities, and positive communication as the most salient factors that contributed to a quality sport experience for youth athletes. Although existing sport programs may aspire to create this quality sport experience among their youth participants, more efforts must be taken to intentionally target each of these factors to create optimal experiences for youth participating within sports. Critically, the findings from this study will help provide a framework for how to effectively capture youth quality sport experiences, and the development of the SEM:CY. Implications of a quantitative survey tool based on these findings will enable sport program administrators to evaluate the quality sport experiences among their youth athletes and provide information about targets for intervention and policy or practices to improve the quality of youth sport experiences.
